# Diaqua­bis(*N*,*N*-diethyl­nicotinamide-κ*N* ^1^)bis­[4-(dimethyl­amino)benzoato-κ*O*]nickel(II)

**DOI:** 10.1107/S1600536809030098

**Published:** 2009-07-31

**Authors:** Tuncer Hökelek, Yasemin Süzen, Barış Tercan, Özgür Aybirdi, Hacali Necefoğlu

**Affiliations:** a Department of Physics, Hacettepe University, 06800 Beytepe, Ankara, Turkey; b Department of Chemistry, Faculty of Science, Anadolu University, 26470 Yenibağlar, Eskişehir, Turkey; c Department of Physics, Karabük University, 78050, Karabük, Turkey; d Department of Chemistry, Kafkas University, 63100 Kars, Turkey

## Abstract

The centrosymmetric title Ni^II^ complex, [Ni(C_9_H_10_NO_2_)_2_(C_10_H_14_N_2_O)_2_(H_2_O)_2_], contains two dimethyl­amino­benzoate (DMAB), two diethyl­nicotinamide (DENA) ligands and two water mol­ecules, all of them monodentate. The four O atoms in the equatorial plane around the Ni^II^ atom form a slightly distorted square-planar arrangement, while the slightly distorted octa­hedral coordination is completed by the two pyridine N atoms of the DENA ligands in axial positions. The Ni^II^ atom is displaced by 0.681 (1) Å out of the least-squares plane of the carboxyl­ate group. The dihedral angle between the carboxyl­ate group and the adjacent benzene ring is 5.61 (7)°, while the pyridine and benzene rings are oriented at a dihedral angle of 73.20 (4)°. An intra­molecular O—H⋯O hydrogen bond results in the formation of a six-membered ring with a twisted conformation. In the crystal structure, inter­molecular O—H⋯O and C—H⋯O hydrogen bonds link mol­ecules into a three-dimensional network. Two weak C—H⋯π inter­actions are also present.

## Related literature

For our ongoing investigations of the transition metal complexes of nicotinamide, and/or the nicotinic acid derivative *N*,*N*-diethyl­nicotinamide, an important respiratory stimulant, see: Bigoli *et al.* (1972 ▶); Krishnamachari (1974 ▶). For related structures, see: Hökelek *et al.* (2009 ▶); Sertçelik *et al.* (2009 ▶).
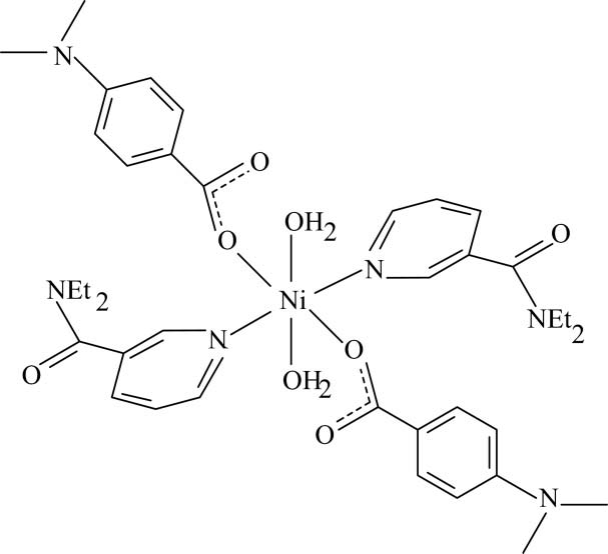


## Experimental

### 

#### Crystal data

[Ni(C_9_H_10_NO_2_)_2_(C_10_H_14_N_2_O)_2_(H_2_O)_2_]
*M* *_r_* = 779.57
Monoclinic, 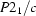

*a* = 6.5081 (1) Å
*b* = 20.3157 (3) Å
*c* = 14.7235 (2) Å
β = 98.487 (2)°
*V* = 1925.37 (5) Å^3^
*Z* = 2
Mo *K*α radiation
μ = 0.56 mm^−1^
*T* = 100 K
0.53 × 0.43 × 0.28 mm

#### Data collection

Bruker Kappa APEXII CCD area-detector diffractometer
Absorption correction: multi-scan (*SADABS*; Bruker, 2005 ▶) *T*_min_ = 0.751, *T*_max_ = 0.851
18748 measured reflections
4819 independent reflections
4332 reflections with *I* > 2σ(*I*)
*R*_int_ = 0.019

#### Refinement

*R*[*F* ^2^ > 2σ(*F* ^2^)] = 0.029
*wR*(*F* ^2^) = 0.076
*S* = 1.04
4819 reflections
253 parameters
H atoms treated by a mixture of independent and constrained refinement
Δρ_max_ = 0.47 e Å^−3^
Δρ_min_ = −0.41 e Å^−3^

### 

Data collection: *APEX2* (Bruker, 2007 ▶); cell refinement: *SAINT* (Bruker, 2007 ▶); data reduction: *SAINT* ; program(s) used to solve structure: *SHELXS97* (Sheldrick, 2008 ▶); program(s) used to refine structure: *SHELXL97* (Sheldrick, 2008 ▶); molecular graphics: *ORTEP-3 for Windows* (Farrugia, 1997 ▶); software used to prepare material for publication: *WinGX* (Farrugia, 1999 ▶) and *PLATON* (Spek, 2009 ▶).

## Supplementary Material

Crystal structure: contains datablocks I, global. DOI: 10.1107/S1600536809030098/xu2570sup1.cif

Structure factors: contains datablocks I. DOI: 10.1107/S1600536809030098/xu2570Isup2.hkl

Additional supplementary materials:  crystallographic information; 3D view; checkCIF report

## Figures and Tables

**Table 1:** Selected bond lengths (Å)

Ni1—O1	2.0498 (8)
Ni1—O4	2.0842 (9)
Ni1—N1	2.0962 (10)

**Table 2:** Hydrogen-bond geometry (Å, °)

*D*—H⋯*A*	*D*—H	H⋯*A*	*D*⋯*A*	*D*—H⋯*A*
O4—H41⋯O2^i^	0.84 (2)	1.97 (2)	2.7875 (12)	163 (2)
O4—H42⋯O2	0.85 (2)	1.82 (2)	2.6552 (12)	165.9 (18)
C11—H11⋯O3^ii^	0.93	2.45	3.3641 (15)	167
C18—H18*A*⋯O3^iii^	0.96	2.48	3.4038 (17)	161
C19—H19*B*⋯*Cg*1^iv^	0.96	2.82	3.7203 (15)	157
C15—H15*A*⋯*Cg*2^v^	0.96	2.91	3.7575 (16)	148

Symmetry codes: (i) 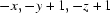; (ii) 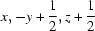; (iii) 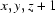; (iv) 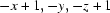; (v) 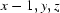. *Cg*1 and *Cg*2 are the centroids of the C2–C7 and N1/C8–C12 rings, respectively.

**Fig. 1.**
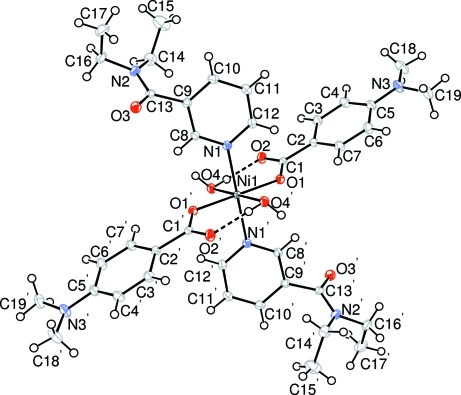
The molecular structure of the title molecule with the atom-numbering scheme. Displacement ellipsoids are drawn at the 50% probability level. Primed atoms are generated by the symmetry operator:(') -x, -y, -z. Dashed lines indicate intramolecular hydrogen bonding.

## supplementary crystallographic information

### Comment

As a part of our ongoing investigation on transition metal complexes of nicotinamide (NA), one form of niacin (Krishnamachari, 1974), and/or the nicotinic acid derivative *N*,*N*-diethylnicotinamide (DENA), an important respiratory stimulant (Bigoli *et al.*, 1972), the title compound was synthesized and its crystal structure is reported herein.

The title compound is a monomeric complex, with Ni^II^ ion on a centre of symmetry, consisting of two DENA and two dimethylaminobenzoate (DMAB) ligands and two water molecules. The structures of similar comlexes of Ni^II^ ion, [Ni(C_8_H_5_O_3_)_2_(C_10_H_14_N_2_O)_2_(H_2_O)_2_] (Sertçelik *et al.*, 2009) and [Ni(C_7_H_4_ClO_2_)_2_(C_10_H_14_N_2_O)_2_(H_2_O)_2_] (Hökelek *et al.*, 2009) have also been determined.

In the title compound, all ligands are monodentate. The four O atoms (O1, O4, and the symmetry-related atoms, O1', O4') in the equatorial plane around the Ni atom form a slightly distorted square-planar arrangement, while the slightly distorted octahedral coordination is completed by the two pyridine N atoms (N1, N1') of the DENA ligands at 2.0962 (10) Å from the Ni atom in the axial positions (Fig. 1 and Table 1). The average Ni—O bond length is 2.0670 (9) Å and the Ni atom is displaced out of the least-squares plane of the carboxylate group (O1/C1/O2) by -0.681 (1) Å. The dihedral angle between the planar carboxylate group and the benzene ring A (C2—C7) is 5.61 (7)°, while that between rings A and B (N1/C8—C12) is 73.20 (4)°. Intramolecular O—H···O hydrogen bond results in the formation of a six-membered ring C (Ni1/O1/O2/O4/C1/H42) having twisted conformation.

In the crystal structure, intermolecular O—H···O and C—H···O hydrogen bonds (Table 2) link the molecules into a three-dimensional network, in which they may be effective in the stabilization of the structure. Two weak C—H···π interactions (Table 1) are also found.

### Experimental

The title compound was prepared by the reaction of NiSO_4_.6H_2_O (1.31 g, 5 mmol) in H_2_O (50 ml) and DENA (1.78 g, 10 mmol) in H_2_O (50 ml) with sodium *p*-dimethylaminobenzoate (1.88 g, 10 mmol) in H_2_O (100 ml). The mixture was filtered and set aside to crystallize at ambient temperature for one week, giving blue single crystals.

### Refinement

Atoms H41 and H42 (for H_2_O) were located in a difference Fourier map and refined isotropically. The remaining H atoms were positioned geometrically with C—H = 0.93, 0.97 and 0.96 Å, for aromatic, methylene and methyl H atoms, respectively, and constrained to ride on their parent atoms, with *U*_iso_(H) = *xU*_eq_(C), where *x* = 1.5 for methyl H and *x* = 1.2 for all other H atoms.

### Crystal data

**Table:**

[Ni(C_9_H_10_NO_2_)_2_(C_10_H_14_N_2_O)_2_(H_2_O)_2_]	*F*(000) = 828
*M**_r_* = 779.57	*D*_x_ = 1.345 Mg m^−^^3^
Monoclinic, *P*2_1_/*c*	Mo *K*α radiation, λ = 0.71073 Å
Hall symbol: -P 2ybc	Cell parameters from 9995 reflections
*a* = 6.5081 (1) Å	θ = 2.4–28.4°
*b* = 20.3157 (3) Å	µ = 0.56 mm^−^^1^
*c* = 14.7235 (2) Å	*T* = 100 K
β = 98.487 (2)°	Block, blue
*V* = 1925.37 (5) Å^3^	0.53 × 0.43 × 0.28 mm
*Z* = 2	

### Data collection

**Table:**

Bruker Kappa APEXII CCD area-detector diffractometer	4819 independent reflections
Radiation source: fine-focus sealed tube	4332 reflections with *I* > 2σ(*I*)
graphite	*R*_int_ = 0.019
φ and ω scans	θ_max_ = 28.4°, θ_min_ = 1.7°
Absorption correction: multi-scan (*SADABS*; Bruker, 2005)	*h* = −8→8
*T*_min_ = 0.751, *T*_max_ = 0.851	*k* = −25→27
18748 measured reflections	*l* = −19→19

### Refinement

**Table:**

Refinement on *F*^2^	Primary atom site location: structure-invariant direct methods
Least-squares matrix: full	Secondary atom site location: difference Fourier map
*R*[*F*^2^ > 2σ(*F*^2^)] = 0.029	Hydrogen site location: inferred from neighbouring sites
*wR*(*F*^2^) = 0.076	H atoms treated by a mixture of independent and constrained refinement
*S* = 1.04	*w* = 1/[σ^2^(*F*_o_^2^) + (0.0382*P*)^2^ + 0.8491*P*] where *P* = (*F*_o_^2^ + 2*F*_c_^2^)/3
4819 reflections	(Δ/σ)_max_ = 0.001
253 parameters	Δρ_max_ = 0.47 e Å^−^^3^
0 restraints	Δρ_min_ = −0.41 e Å^−^^3^

### Special details

**Table:**

Geometry. All e.s.d.'s (except the e.s.d. in the dihedral angle between two l.s. planes) are estimated using the full covariance matrix. The cell e.s.d.'s are taken into account individually in the estimation of e.s.d.'s in distances, angles and torsion angles; correlations between e.s.d.'s in cell parameters are only used when they are defined by crystal symmetry. An approximate (isotropic) treatment of cell e.s.d.'s is used for estimating e.s.d.'s involving l.s. planes.
Refinement. Refinement of *F*^2^ against ALL reflections. The weighted *R*-factor *wR* and goodness of fit *S* are based on *F*^2^, conventional *R*-factors *R* are based on *F*, with *F* set to zero for negative *F*^2^. The threshold expression of *F*^2^ > σ(*F*^2^) is used only for calculating *R*-factors(gt) *etc*. and is not relevant to the choice of reflections for refinement. *R*-factors based on *F*^2^ are statistically about twice as large as those based on *F*, and *R*- factors based on ALL data will be even larger.

### Fractional atomic coordinates and isotropic or equivalent isotropic displacement parameters (Å^2^)

**Table:**

	*x*	*y*	*z*	*U*_iso_*/*U*_eq_	
Ni1	0.5000	0.5000	0.5000	0.01159 (6)	
O1	0.46213 (13)	0.50364 (4)	0.63567 (6)	0.01499 (17)	
O2	0.12365 (13)	0.52742 (4)	0.62891 (5)	0.01770 (17)	
O3	0.24871 (14)	0.28574 (5)	0.24645 (6)	0.02031 (19)	
O4	0.21205 (14)	0.54434 (4)	0.46008 (6)	0.01635 (17)	
H41	0.122 (3)	0.5242 (11)	0.4234 (13)	0.039 (5)*	
H42	0.169 (3)	0.5441 (9)	0.5121 (14)	0.039 (5)*	
N1	0.37272 (15)	0.40504 (5)	0.48904 (6)	0.01379 (19)	
N2	−0.07839 (16)	0.27925 (5)	0.28157 (7)	0.0179 (2)	
N3	0.31666 (19)	0.39407 (6)	1.02652 (7)	0.0242 (2)	
C1	0.29426 (19)	0.50566 (5)	0.66964 (7)	0.0135 (2)	
C2	0.30181 (18)	0.47867 (6)	0.76460 (7)	0.0145 (2)	
C3	0.12187 (19)	0.47318 (6)	0.80487 (8)	0.0177 (2)	
H3	−0.0035	0.4882	0.7731	0.021*	
C4	0.1257 (2)	0.44578 (6)	0.89121 (8)	0.0197 (2)	
H4	0.0029	0.4423	0.9161	0.024*	
C5	0.3124 (2)	0.42317 (6)	0.94177 (8)	0.0182 (2)	
C6	0.4949 (2)	0.43026 (7)	0.90178 (8)	0.0204 (2)	
H6	0.6216	0.4170	0.9342	0.024*	
C7	0.48766 (19)	0.45671 (6)	0.81467 (8)	0.0177 (2)	
H7	0.6096	0.4599	0.7890	0.021*	
C8	0.28565 (18)	0.38146 (6)	0.40728 (7)	0.0142 (2)	
H8	0.2801	0.4084	0.3559	0.017*	
C9	0.20358 (18)	0.31856 (6)	0.39614 (7)	0.0141 (2)	
C10	0.21297 (19)	0.27802 (6)	0.47281 (8)	0.0174 (2)	
H10	0.1598	0.2355	0.4673	0.021*	
C11	0.30290 (19)	0.30200 (6)	0.55766 (8)	0.0176 (2)	
H11	0.3104	0.2760	0.6100	0.021*	
C12	0.38123 (18)	0.36540 (6)	0.56268 (7)	0.0158 (2)	
H12	0.4425	0.3813	0.6195	0.019*	
C13	0.12500 (19)	0.29353 (6)	0.30122 (8)	0.0149 (2)	
C14	−0.2321 (2)	0.29609 (7)	0.34147 (9)	0.0241 (3)	
H14A	−0.3490	0.3180	0.3053	0.029*	
H14B	−0.1700	0.3268	0.3880	0.029*	
C15	−0.3107 (2)	0.23647 (9)	0.38829 (12)	0.0381 (4)	
H15A	−0.3958	0.2508	0.4325	0.057*	
H15B	−0.1947	0.2119	0.4188	0.057*	
H15C	−0.3912	0.2091	0.3432	0.057*	
C16	−0.1508 (2)	0.24500 (7)	0.19478 (9)	0.0234 (3)	
H16A	−0.0881	0.2653	0.1459	0.028*	
H16B	−0.3002	0.2500	0.1800	0.028*	
C17	−0.0974 (2)	0.17227 (7)	0.19917 (10)	0.0292 (3)	
H17A	−0.1409	0.1525	0.1402	0.044*	
H17B	−0.1673	0.1513	0.2444	0.044*	
H17C	0.0500	0.1670	0.2157	0.044*	
C18	0.1327 (3)	0.39476 (7)	1.07082 (9)	0.0301 (3)	
H18A	0.1616	0.3730	1.1292	0.045*	
H18B	0.0219	0.3723	1.0328	0.045*	
H18C	0.0924	0.4395	1.0799	0.045*	
C19	0.5140 (2)	0.37889 (7)	1.08181 (9)	0.0273 (3)	
H19A	0.4902	0.3617	1.1401	0.041*	
H19B	0.5962	0.4182	1.0913	0.041*	
H19C	0.5863	0.3468	1.0507	0.041*	

### Atomic displacement parameters (Å^2^)

**Table:**

	*U*^11^	*U*^22^	*U*^33^	*U*^12^	*U*^13^	*U*^23^
Ni1	0.01140 (11)	0.01277 (11)	0.01029 (10)	−0.00191 (7)	0.00061 (7)	0.00090 (7)
O1	0.0140 (4)	0.0182 (4)	0.0128 (4)	−0.0029 (3)	0.0018 (3)	0.0004 (3)
O2	0.0151 (4)	0.0226 (4)	0.0150 (4)	0.0013 (3)	0.0009 (3)	0.0008 (3)
O3	0.0219 (5)	0.0234 (5)	0.0160 (4)	−0.0044 (4)	0.0040 (3)	−0.0023 (3)
O4	0.0145 (4)	0.0197 (4)	0.0143 (4)	−0.0009 (3)	0.0006 (3)	0.0025 (3)
N1	0.0130 (5)	0.0144 (5)	0.0139 (4)	−0.0008 (4)	0.0018 (3)	0.0009 (3)
N2	0.0162 (5)	0.0170 (5)	0.0192 (5)	−0.0022 (4)	−0.0016 (4)	−0.0012 (4)
N3	0.0285 (6)	0.0277 (6)	0.0172 (5)	0.0012 (5)	0.0058 (4)	0.0072 (4)
C1	0.0165 (5)	0.0112 (5)	0.0125 (5)	−0.0029 (4)	0.0012 (4)	−0.0020 (4)
C2	0.0171 (6)	0.0140 (5)	0.0125 (5)	−0.0016 (4)	0.0027 (4)	−0.0008 (4)
C3	0.0159 (6)	0.0204 (6)	0.0168 (5)	0.0008 (5)	0.0024 (4)	0.0011 (4)
C4	0.0189 (6)	0.0223 (6)	0.0194 (5)	−0.0004 (5)	0.0077 (4)	0.0022 (4)
C5	0.0245 (6)	0.0159 (5)	0.0147 (5)	−0.0004 (5)	0.0042 (4)	0.0010 (4)
C6	0.0187 (6)	0.0242 (6)	0.0177 (5)	0.0030 (5)	0.0009 (4)	0.0038 (4)
C7	0.0157 (6)	0.0208 (6)	0.0174 (5)	0.0005 (5)	0.0044 (4)	0.0021 (4)
C8	0.0134 (5)	0.0156 (5)	0.0135 (5)	−0.0004 (4)	0.0021 (4)	0.0019 (4)
C9	0.0129 (5)	0.0153 (5)	0.0142 (5)	−0.0001 (4)	0.0018 (4)	−0.0004 (4)
C10	0.0194 (6)	0.0138 (5)	0.0193 (5)	−0.0021 (4)	0.0036 (4)	0.0017 (4)
C11	0.0202 (6)	0.0174 (6)	0.0154 (5)	0.0007 (5)	0.0030 (4)	0.0041 (4)
C12	0.0160 (6)	0.0183 (6)	0.0129 (5)	0.0009 (4)	0.0009 (4)	0.0010 (4)
C13	0.0179 (6)	0.0103 (5)	0.0158 (5)	−0.0018 (4)	−0.0003 (4)	0.0014 (4)
C14	0.0156 (6)	0.0278 (7)	0.0285 (6)	0.0027 (5)	0.0018 (5)	0.0014 (5)
C15	0.0214 (7)	0.0479 (10)	0.0471 (9)	0.0047 (7)	0.0118 (6)	0.0191 (7)
C16	0.0233 (7)	0.0214 (6)	0.0223 (6)	−0.0049 (5)	−0.0066 (5)	−0.0020 (5)
C17	0.0324 (8)	0.0206 (7)	0.0331 (7)	−0.0061 (6)	−0.0004 (6)	−0.0057 (5)
C18	0.0427 (9)	0.0274 (7)	0.0238 (6)	0.0071 (6)	0.0174 (6)	0.0082 (5)
C19	0.0373 (8)	0.0272 (7)	0.0166 (5)	0.0057 (6)	0.0016 (5)	0.0050 (5)

### Geometric parameters (Å, °)

**Table:**

Ni1—O1	2.0498 (8)	C8—H8	0.9300
Ni1—O1^i^	2.0498 (8)	C9—C8	1.3853 (16)
Ni1—O4	2.0842 (9)	C9—C10	1.3915 (15)
Ni1—O4^i^	2.0842 (9)	C9—C13	1.5042 (15)
Ni1—N1	2.0962 (10)	C10—H10	0.9300
Ni1—N1^i^	2.0962 (10)	C11—C10	1.3874 (16)
O1—C1	1.2675 (14)	C11—C12	1.3831 (17)
O2—C1	1.2615 (15)	C11—H11	0.9300
O3—C13	1.2309 (15)	C12—H12	0.9300
O4—H41	0.84 (2)	C14—H14A	0.9700
O4—H42	0.85 (2)	C14—H14B	0.9700
N1—C8	1.3411 (14)	C15—C14	1.519 (2)
N1—C12	1.3452 (14)	C15—H15A	0.9600
N2—C13	1.3443 (16)	C15—H15B	0.9600
N2—C14	1.4682 (16)	C15—H15C	0.9600
N3—C18	1.4457 (17)	C16—N2	1.4700 (15)
N3—C19	1.4485 (18)	C16—H16A	0.9700
C2—C1	1.4958 (15)	C16—H16B	0.9700
C2—C7	1.3937 (17)	C17—C16	1.5173 (19)
C3—C2	1.3930 (16)	C17—H17A	0.9600
C3—C4	1.3846 (16)	C17—H17B	0.9600
C3—H3	0.9300	C17—H17C	0.9600
C4—H4	0.9300	C18—H18A	0.9600
C5—N3	1.3773 (15)	C18—H18B	0.9600
C5—C4	1.4050 (18)	C18—H18C	0.9600
C5—C6	1.4086 (17)	C19—H19A	0.9600
C6—C7	1.3850 (16)	C19—H19B	0.9600
C6—H6	0.9300	C19—H19C	0.9600
C7—H7	0.9300		
			
O1—Ni1—O1^i^	180.000 (1)	C8—C9—C10	118.69 (10)
O1—Ni1—O4	91.54 (3)	C8—C9—C13	119.61 (10)
O1^i^—Ni1—O4	88.46 (3)	C10—C9—C13	121.45 (10)
O1—Ni1—O4^i^	88.46 (3)	C9—C10—H10	120.5
O1^i^—Ni1—O4^i^	91.54 (3)	C11—C10—C9	118.99 (11)
O1—Ni1—N1	90.30 (3)	C11—C10—H10	120.5
O1^i^—Ni1—N1	89.70 (3)	C10—C11—H11	120.7
O1—Ni1—N1^i^	89.70 (3)	C12—C11—C10	118.58 (10)
O1^i^—Ni1—N1^i^	90.30 (3)	C12—C11—H11	120.7
O4—Ni1—O4^i^	180.0	N1—C12—C11	122.91 (11)
O4—Ni1—N1	92.72 (4)	N1—C12—H12	118.5
O4^i^—Ni1—N1	87.28 (4)	C11—C12—H12	118.5
O4—Ni1—N1^i^	87.28 (4)	O3—C13—N2	123.34 (11)
O4^i^—Ni1—N1^i^	92.72 (4)	O3—C13—C9	119.12 (11)
N1—Ni1—N1^i^	180.0	N2—C13—C9	117.51 (10)
C1—O1—Ni1	128.34 (8)	N2—C14—C15	113.05 (12)
Ni1—O4—H41	118.7 (14)	N2—C14—H14A	109.0
Ni1—O4—H42	98.5 (13)	N2—C14—H14B	109.0
H41—O4—H42	106.5 (18)	C15—C14—H14A	109.0
C8—N1—C12	118.12 (10)	C15—C14—H14B	109.0
C8—N1—Ni1	120.51 (7)	H14A—C14—H14B	107.8
C12—N1—Ni1	121.34 (8)	C14—C15—H15A	109.5
C13—N2—C14	123.83 (10)	C14—C15—H15B	109.5
C13—N2—C16	117.93 (10)	C14—C15—H15C	109.5
C14—N2—C16	118.24 (11)	H15A—C15—H15B	109.5
C5—N3—C18	119.82 (11)	H15A—C15—H15C	109.5
C5—N3—C19	119.89 (11)	H15B—C15—H15C	109.5
C18—N3—C19	118.24 (11)	N2—C16—C17	112.45 (11)
O1—C1—C2	116.46 (10)	N2—C16—H16A	109.1
O2—C1—O1	124.98 (10)	N2—C16—H16B	109.1
O2—C1—C2	118.55 (10)	C17—C16—H16A	109.1
C3—C2—C1	120.90 (11)	C17—C16—H16B	109.1
C3—C2—C7	117.88 (10)	H16A—C16—H16B	107.8
C7—C2—C1	121.20 (10)	C16—C17—H17A	109.5
C2—C3—H3	119.3	C16—C17—H17B	109.5
C4—C3—C2	121.37 (11)	C16—C17—H17C	109.5
C4—C3—H3	119.3	H17A—C17—H17B	109.5
C3—C4—C5	121.00 (11)	H17A—C17—H17C	109.5
C3—C4—H4	119.5	H17B—C17—H17C	109.5
C5—C4—H4	119.5	N3—C18—H18A	109.5
N3—C5—C4	121.38 (11)	N3—C18—H18B	109.5
N3—C5—C6	121.13 (12)	N3—C18—H18C	109.5
C4—C5—C6	117.48 (10)	H18A—C18—H18B	109.5
C5—C6—H6	119.6	H18A—C18—H18C	109.5
C7—C6—C5	120.76 (11)	H18B—C18—H18C	109.5
C7—C6—H6	119.6	N3—C19—H19A	109.5
C2—C7—H7	119.3	N3—C19—H19B	109.5
C6—C7—C2	121.48 (11)	N3—C19—H19C	109.5
C6—C7—H7	119.3	H19A—C19—H19B	109.5
N1—C8—C9	122.70 (10)	H19A—C19—H19C	109.5
N1—C8—H8	118.7	H19B—C19—H19C	109.5
C9—C8—H8	118.7		
			
O4—Ni1—O1—C1	24.97 (9)	C7—C2—C1—O2	−177.04 (11)
O4^i^—Ni1—O1—C1	−155.03 (9)	C1—C2—C7—C6	−178.48 (11)
N1—Ni1—O1—C1	−67.76 (9)	C3—C2—C7—C6	−0.08 (18)
N1^i^—Ni1—O1—C1	112.24 (9)	C4—C3—C2—C1	177.32 (11)
O1—Ni1—N1—C8	156.38 (9)	C4—C3—C2—C7	−1.09 (18)
O1^i^—Ni1—N1—C8	−23.62 (9)	C2—C3—C4—C5	0.7 (2)
O4—Ni1—N1—C8	64.83 (9)	C4—C5—N3—C18	−8.82 (19)
O4^i^—Ni1—N1—C8	−115.17 (9)	C4—C5—N3—C19	−172.22 (12)
O1—Ni1—N1—C12	−25.63 (9)	C6—C5—N3—C18	172.36 (13)
O1^i^—Ni1—N1—C12	154.37 (9)	C6—C5—N3—C19	8.97 (19)
O4—Ni1—N1—C12	−117.18 (9)	N3—C5—C4—C3	−178.04 (12)
O4^i^—Ni1—N1—C12	62.82 (9)	C6—C5—C4—C3	0.81 (19)
Ni1—O1—C1—O2	−25.08 (16)	N3—C5—C6—C7	176.89 (12)
Ni1—O1—C1—C2	153.63 (8)	C4—C5—C6—C7	−1.96 (19)
Ni1—N1—C8—C9	178.55 (8)	C5—C6—C7—C2	1.6 (2)
C12—N1—C8—C9	0.49 (17)	C10—C9—C8—N1	−0.41 (18)
Ni1—N1—C12—C11	−178.58 (9)	C13—C9—C8—N1	−174.79 (11)
C8—N1—C12—C11	−0.54 (17)	C8—C9—C10—C11	0.36 (17)
C14—N2—C13—O3	−171.82 (11)	C13—C9—C10—C11	174.63 (11)
C14—N2—C13—C9	9.85 (17)	C8—C9—C13—O3	64.89 (15)
C16—N2—C13—O3	7.90 (18)	C8—C9—C13—N2	−116.71 (12)
C16—N2—C13—C9	−170.42 (10)	C10—C9—C13—O3	−109.33 (13)
C13—N2—C14—C15	−108.56 (14)	C10—C9—C13—N2	69.07 (15)
C16—N2—C14—C15	71.71 (15)	C12—C11—C10—C9	−0.40 (18)
C3—C2—C1—O1	−174.19 (11)	C10—C11—C12—N1	0.51 (18)
C3—C2—C1—O2	4.60 (17)	C17—C16—N2—C13	76.55 (15)
C7—C2—C1—O1	4.16 (16)	C17—C16—N2—C14	−103.71 (14)

Symmetry codes: (i) −*x*+1, −*y*+1, −*z*+1.

### Hydrogen-bond geometry (Å, °)

**Table:**

*D*—H···*A*	*D*—H	H···*A*	*D*···*A*	*D*—H···*A*
O4—H41···O2^ii^	0.84 (2)	1.97 (2)	2.7875 (12)	163 (2)
O4—H42···O2	0.85 (2)	1.82 (2)	2.6552 (12)	165.9 (18)
C11—H11···O3^iii^	0.93	2.45	3.3641 (15)	167
C18—H18A···O3^iv^	0.96	2.48	3.4038 (17)	161
C19—H19B···Cg1^v^	0.96	2.82	3.7203 (15)	157
C15—H15A···Cg2^vi^	0.96	2.91	3.7575 (16)	148

Symmetry codes: (ii) −*x*, −*y*+1, −*z*+1; (iii) *x*, −*y*+1/2, *z*+1/2; (iv) *x*, *y*, *z*+1; (v) −*x*+1, −*y*, −*z*+1; (vi) *x*−1, *y*, *z*.
